# Transgenic Rice Seed Extracts Exert Immunomodulatory Effects by Modulating Immune-Related Biomarkers in RAW264.7 Macrophage Cells

**DOI:** 10.3390/nu14194143

**Published:** 2022-10-05

**Authors:** Chaiwat Monmai, Jin-Suk Kim, So-Hyeon Baek

**Affiliations:** Department of Agricultural Life Science, Sunchon National University, Suncheon 59722, Korea

**Keywords:** transgenic rice, protopanaxadiol, PPD, immune enhancement, anti-inflammation, macrophage cells, NF-κB, MAPK

## Abstract

Protopanaxadiol (PPD), a native active triterpenoid present in *Panax ginseng*, has been reported to exert immune-related effects. We previously created PPD-producing transgenic rice by introducing the *P. ginseng* protopanaxadiol synthase and dammarenediol-II synthase genes into Dongjin rice. In the present study, the seeds of the T_4_ generation of this transgenic rice were tested for their immunomodulatory effects in RAW264.7 macrophage cells. Treatment with transgenic rice seed extract in RAW264.7 cells (i) significantly enhanced nitric oxide (NO) production in a dose-dependent manner without any cytotoxicity (up to 100 µg/mL), (ii) upregulated the expression of immune-related genes and increased production of the inflammation mediator prostaglandin E_2_ (PGE_2_), and (iii) activated nuclear factor-κB (NF-κB) and mitogen-activated protein kinase (MAPK) by promoting the phosphorylation of NF-κB p65, p38 MAPK, and c-Jun N-terminal protein kinase (JNK). In lipopolysaccharide (LPS)-treated RAW264.7 cells used to mimic the inflammation condition, treatment with transgenic rice seed extract significantly reduced NO production, proinflammatory cytokine expression, and PGE_2_ production, all of which are LPS-induced inflammation biomarkers, by inhibiting the phosphorylation of NF-κB p65, p38 MAPK, and JNK. Collectively, these results indicate that PPD-producing transgenic rice has immunomodulatory effects.

## 1. Introduction

The immune system in the host cells plays an important role in defense against pathogens and various other stimuli. Depending on the functional activities, it is divided into two groups—innate and adaptive immune responses [1,2]. Macrophages, which are active in both types of immune responses, offer protection against foreign microorganisms through phagocytosis as well as the release of proinflammatory cytokines and mediators, e.g., interleukin (IL), tumor necrosis factor (TNF), and nitric oxide (NO) [3,4,5]. Macrophages can exhibit different phenotypes depending on their activation mode [6]. Based on their polarization, they are mainly categorized as classical (M1) and alternative (M2) macrophages [7]. M1 macrophage is associated with an inflammatory microenvironment, whereas M2 macrophage is associated with an anti-inflammatory microenvironment [8]. Immune protection against pathogens also involves the activation of various signaling pathways, including mitogen-activated protein kinase (MAPK) and nuclear factor-κB (NF-κB) pathways [2].

Ginseng (*Panax ginseng* Meyer) is widely used as a natural pharmaceutical herb worldwide, particularly in Asia [9]. Ginseng is recognized for its potential to enhance immune responses, homeostasis, and self-healing [10,11]. The active compounds isolated from ginseng include ginsenosides [12], polyacetylenes [13], polysaccharides [14], and phenolics [15]. Ginsenoside is the main active compound of ginseng and can be divided into two major groups: protopanaxadiol (PPD) and protopanaxtriol glycosides [8]. Several studies have reported the immune-related effects of PPD, which include anticancer [16,17,18], antistress [19], immune boosting [20,21], and anti-inflammatory effects [19,22,23].

We previously created transgenic rice that overexpresses the *P. ginseng* dammarenediol-II synthase and protopanaxadiol synthase genes [24]. This transgenic rice produces PPD and dammarenediol-II, which are not found in normal [Dongjin (DJ)] rice. In the present study, we evaluated the immune-related effects of the seed extract of this transgenic rice in RAW264.7 macrophage cells with and without lipopolysaccharide (LPS) induction (used to mimic inflammation). Overall, our study shows that PPD-producing transgenic rice exerts immunomodulatory effects in RAW264.7 macrophage cells.

## 2. Materials and Methods

### 2.1. Sample Extract Preparation

Brown rice grains were collected and ground using a blender. To prepare sample extracts, 5 g of each sample powder was added to 100% methanol and sonicated for 1 h. The mixture sample was then filtered through 5 µm filter paper (Hyundai Micro, Seoul, Korea). The filtered solution was concentrated by removing the solvent using a rotary evaporator at 50 °C, and lyophilization was used to collect the crude extract. Crude extracts were adjusted to concentrations of 10, 25, 50, and 100 mg/mL using dimethyl sulfoxide (DMSO) in preparation for an in vitro immunomodulatory assay.

### 2.2. RAW264.7 Macrophage Culture

RAW264.7 cells were obtained from Korean Cell Line Bank (Seoul, Korea) and cultured in RPMI-1640 medium. The culture medium contained fetal bovine serum (10%) and antibiotics [penicillin/streptomycin (1%)]. The cells were incubated in a humidity-controlled incubator at 37 °C with 5% CO_2_ and maintained through weekly cell passaging.

### 2.3. NO Production and Cell Viability Assays

Cells (1 × 10^5^ cells/well) were seeded and incubated at 37 °C for 24 h. The culture medium was replaced with various concentrations of the rice seed extract treatments. After 1 h of pretreatment, some cells received LPS (1 µg/mL) stimulation, whereas some cells received no stimulation, and the assay plate was incubated for a further 24 h. NO production was evaluated using Griess reagent (Sigma-Aldrich, St. Louis, MO, USA) and quantified by constructing a standard curve of sodium nitrite (NaNO_2_). An EZ-Cytox Cell Viability Assay Kit was used to measure the cytotoxicity of the crude extracts. Cell viability was calculated based on the following formula:

$$\mathrm{Cell}\;\mathrm{viability}\;\left(\%\right)=\frac{A_{450}{\;\mathrm{of}\;\mathrm{treatment}\;-\;A}_{450}\;\mathrm{of}\;\mathrm{blank}\;}{A_{450}{\;\mathrm{of}\;\mathrm{control}\;-\;A}_{450}\;\mathrm{of}\;\mathrm{blank}}\times 100,$$

where A_450_ represents the absorbance at 450 nm and “control” represents the nontreatment group.

### 2.4. RNA Extraction and cDNA Synthesis

The cells with or without LPS stimulation were harvested after 6 h. TRI reagent™ (Invitrogen, Waltham, MA, USA) was used to extract total RNA, and 100% isopropanol was used to precipitate RNA. RNA was collected via centrifugation at 13,000 rpm and 4 °C for 10 min. The RNA pellet obtained was washed with 70% EtOH. After drying, the total RNA pellet was resuspended in 20 µL of nuclease-free water. Total RNA was quantified using a SpectraMax^®^ ABS Plus Microplate Reader (Molecular Devices, San Jose, CA, USA), and cDNA was synthesized using 500 ng of this RNA and a Power cDNA Synthesis Kit (Intron Biotechnology, Seongnam-Si, Korea), according to manufacturer’s instructions.

### 2.5. Quantification of the mRNA Expression of Immune-Related Genes Using Real-Time Quantitative Polymerase Chain Reaction (qPCR)

The mRNA expression of immune-related genes was evaluated using RealMOD™ Green W^2^ 2× qPCR Mix (Intron Biotechnology, Seongnam-Si, Korea) in a CFX Connect Real-Time PCR System (Bio-Rad, Hercules, CA, USA). The PCR reaction included 5 ng of cDNA template and 0.375 µM of each specific primer and was conducted as follows: predenaturation (95 °C for 10 min), 40 cycles of PCR (denaturation: 95 °C for 20 s; annealing: 60 °C for 20 s; extension: 72 °C for 30 s), and final extension (72 °C for 5 min). The sequences of *IL-1β*, *IL-6*, *COX-2*, *iNOS*, *TNF-α*, and *β-actin* were used as described by Monmai et al. [25]; moreover, the sequences of *TLR-4* (NM_021297.3; forward: 5′-CGC TCT GGC ATC ATC TTC AT-3’; reverse: 5′-GTT GCC GTT TCT TGT TCT TCC-3′) were used. The results were analyzed using CFX Maestro software, and *β-actin* was used as the reference gene.

### 2.6. Prostaglandin E_2_ Quantification

The supernatant was collected via centrifugation at 3000 rpm for 10 min, and prostaglandin E_2_ (PGE_2_) production was evaluated using a PGE_2_ ELISA Kit (Enzo Life Sciences, Farmingdale, NY, USA) according to the manufacturer’s instructions. This experiment was performed twice, and PGE_2_ production was calculated based on a standard curve.

### 2.7. Phagocytosis Assay

The phagocytosis activity of RAW264.7 cells was evaluated using a neutral red uptake method [26] with slight modifications. The cells were treated with transgenic rice seed extract samples at 100 µg/mL or with 1 µg/mL of LPS. After 24 h of incubation, the culture medium was removed, and the cells were washed with 1× phosphate-buffered saline (PBS). Neutral red (0.075%; Sigma-Aldrich, St. Louis, MO, USA) was added to each well, and the plate was incubated at room temperature for 2 h. The cells were then washed five times with 1× PBS to remove excess dye and dried. Images of the cells were captured under an IM-3 Series microscope (Optika, Bergamo, Italy). Subsequently, lysis solution [50% EtOH:glacial acetic acid (1:1)] was added to each well. After 2 h of lysis, the absorbance was measured at 540 nm.

### 2.8. Western Blot Assay

Cells were lysed on ice for 30 min with radioimmunoprecipitation assay buffer (Geneall Biotechnology, Seoul, Korea) supplemented with 1× Protease Inhibitor Cocktail Kit 5 (Bio-Medical Science Co., Ltd., Seoul, Korea). Proteins were collected via centrifugation at 13,000 rpm and 4 °C for 30 min, and their concentrations were quantified using Bradford reagent (Sigma-Aldrich, St. Louis, MO, USA) and compared with a bovine serum albumin standard curve. An equal amount (30 µg) of protein sample from each treatment was separated using 10% sodium dodecyl-sulfate polyacrylamide gel electrophoresis and transferred to a nitrocellulose membrane. The membranes were incubated with antibodies specific to the phosphorylation of NF-κB p-65, p38 MAPK, and c-Jun N-terminal protein kinase (JNK) (Cell Signaling, Danvers, MA, USA). Glyceraldehyde 3-phosphate dehydrogenase (GAPDH; Santa Cruz Biotechnology, Dallas, TX, USA) was used as the protein loading control. Protein signaling was detected using Clarity™ Western ECL Substrate (Bio-Rad, Hercules, CA, USA), and the detected signals were imaged and quantified in terms of intensity using a ChemiDoc Imaging System (Bio-Rad, Hercules, CA, USA).

### 2.9. PPD Quantification Using Liquid Chromatography–Mass Spectrometry (LC–MS)

**Figure 1:**
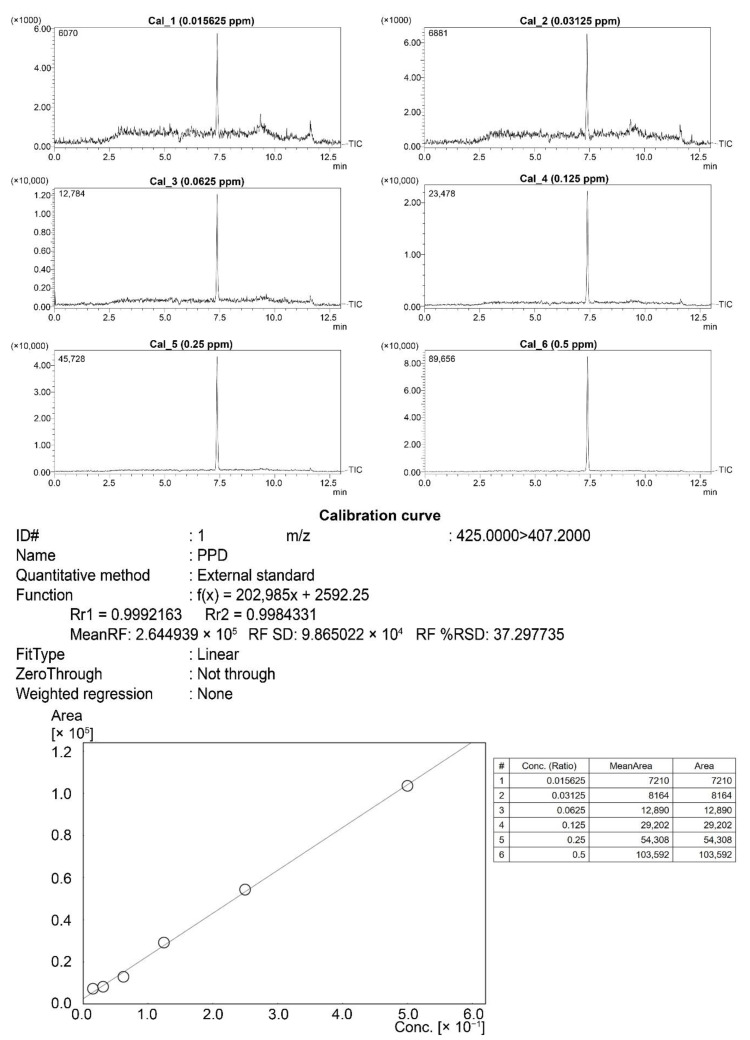
The calibration curve of PPD over the concentration range of 0.0156–0.50000 ppm.

Brown rice grain powder was mixed with 100% methanol and sonicated at 40 °C for 30 min. The mixture was then centrifuged at 13,000 rpm for 30 min, and the supernatant was filtered through a SepPak C-18 Cartridge (Waters, Milford, MA, USA) before LC–MS injection. The calibration curve was constructed using six calibration standard samples of PPD (Figure 1) that included a concentration range of 0.01562–0.50000 ppm. The amount of PPD in each treatment group was quantified using the area under the curve comparison with the calibration curve.

### 2.10. Statistical Analysis

Data are shown as means ± standard deviations. Statistix (Version 8.1; Statistix, Tallahassee, FL, USA) was used to conduct statistical analysis. Data were analyzed using one-way analysis of variance followed by post hoc Duncan’s multiple range tests. The differences between two groups were assessed using t-tests (*p* < 0.05).

## 3. Results

### 3.1. Viability and NO Production of LPS-Stimulated RAW264.7 Cells Incubated with Transgenic Rice Seed Extracts

**Figure 2:**
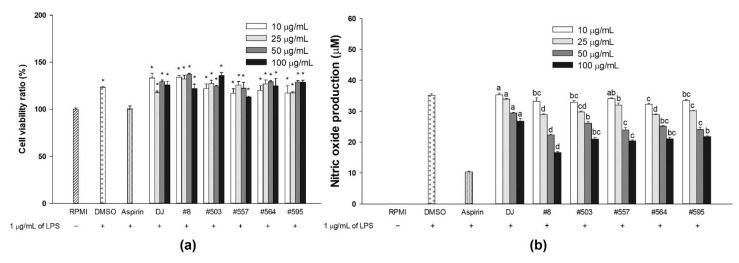
Effects of transgenic rice seed extracts on LPS-stimulated RAW264.7 cells. Effects on (**a**) cell viability and (**b**) NO production. The concentration of DMSO and aspirin were 0.1% and 200 µg/mL, respectively. Data are presented as means ± standard deviations. Significant differences at *p* value of <0.05 (*) were determined via comparisons with the RPMI group. Lowercase letters (a, b, c, and d) indicate significant differences at *p* value of <0.05 among treatments at the same concentration.

To evaluate the effects of the transgenic rice seed extracts on cell viability, RAW264.7 cells were pretreated with various concentrations of each sample and stimulated with 1 µg/mL of LPS. Compared with no treatment [nontreated (RPMI) cells], treatment with transgenic rice seed extract (up to 100 µg/mL) did not exert cytotoxic effects on LPS-stimulated RAW264.7 cells (Figure 2a). Moreover, the transgenic rice seed extracts exerted proliferative effects on the cells at 10–100 µg/mL. Similar results were observed in the DMSO group; however, no significant difference was observed between the aspirin and RPMI groups.

The inhibition of LPS-induced NO production was assessed using the Griess reagent. The increasing concentration of transgenic rice seed extract resulted in significant suppression of NO production in RAW264.7 cells (Figure 2b). Treatment with sample #8 at 100 µg/mL led to the highest suppression of NO production among the transgenic rice seed extracts.

### 3.2. Viability and NO Production of RAW264.7 Cells Incubated with Transgenic Rice Seed Extracts

**Figure 3:**
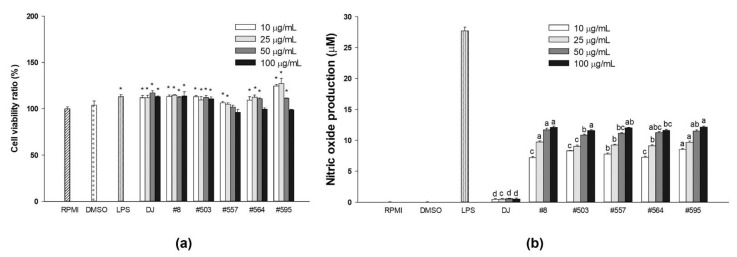
Effects of transgenic rice extracts on RAW264.7 cells without LPS stimulation. Effects on (**a**) cell viability and (**b**) NO production. The concentrations of DMSO and LPS were 0.1% and 1 µg/mL, respectively. Data are presented as means ± standard deviations. Significant differences at *p* value of <0.05 (*) were determined via comparisons with the RPMI group. Lowercase letters (a, b, c, and d) indicate significant differences at *p* value of <0.05 among treatments at the same concentration.

The cytotoxicity of transgenic rice seed extracts was also determined in RAW264.7 cells without LPS treatment (Figure 3). Treatments of each sample up to 100 µg/mL did not cause cytotoxicity in the cells; compared with no treatment [nontreated (RPMI) group], treatment with transgenic rice seed extracts was able to enhance cell proliferation (Figure 3a). However, for samples #557, #564, and #595, cell viability declined when the cells were treated with higher concentrations (50 and 100 µg/mL), although the cell viability of these groups did not differ significantly from that observed in the RPMI group. Compared with no treatment, treatment with DJ rice seed extract increased NO production (Figure 3b). In the group treated with transgenic rice seed extracts, NO production significantly increased with the increase in the concentration of the extract.

### 3.3. Effects on the mRNA Expression Levels of Immune-Associated Genes in LPS-Stimulated RAW264.7 Cells

**Figure 4:**
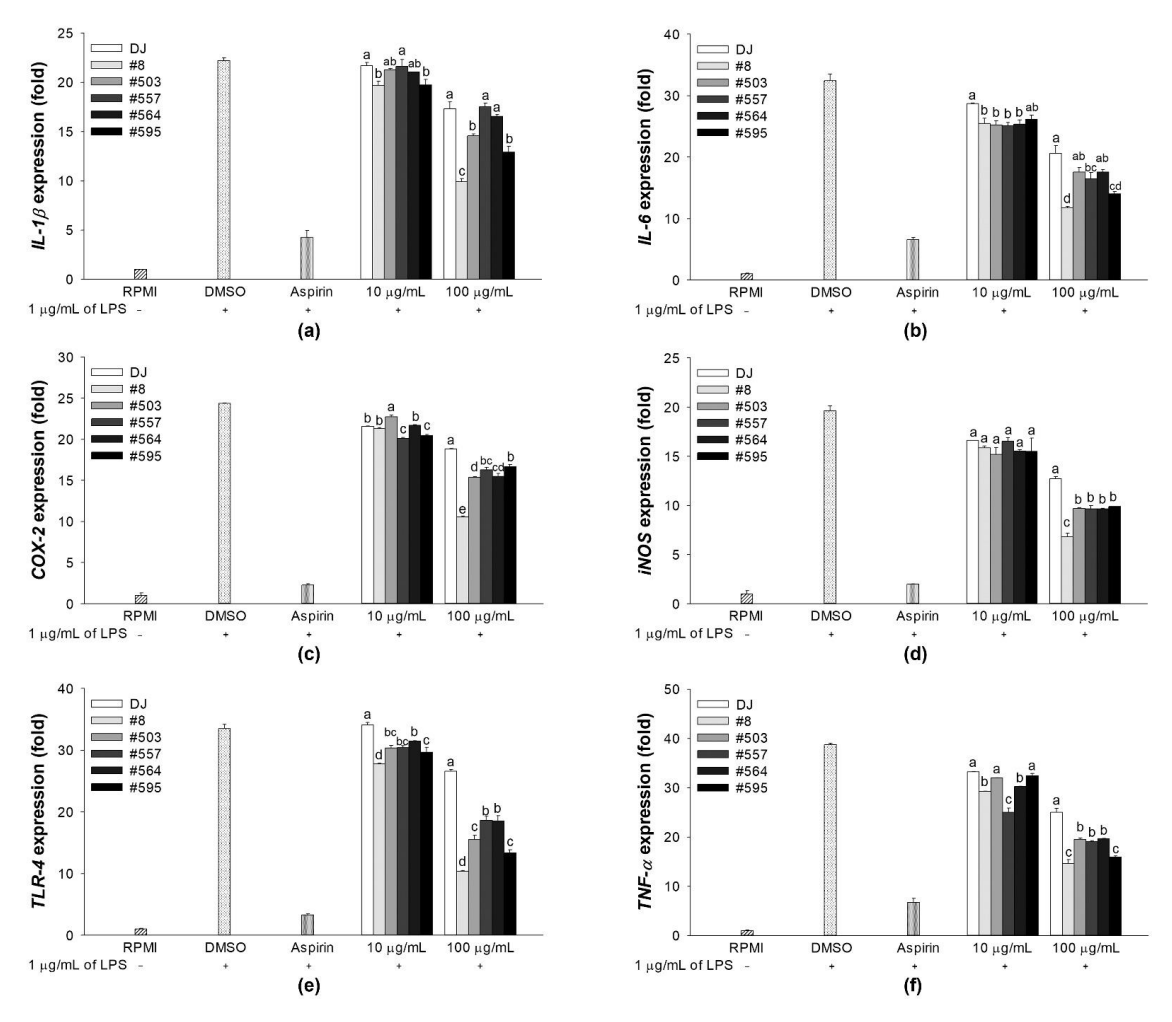
Effects of transgenic rice seed extracts on the expression of immune-associated genes in LPS-stimulated RAW264.7 cells. The expression levels of (**a**) *IL-1β*, (**b**) *IL-6*, (**c**) *COX-2*, (**d***) iNOS*, (**e**) *TLR-4*, and (**f**) *TNF-α* expression levels are shown. The concentrations of DMSO and aspirin were 0.1% and 200 µg/mL, respectively. Data are presented as means ± standard deviations. Lowercase letters (a, b, c, and d) indicate significant differences at *p* < 0.05 among treatments at the same concentration.

Treatment with LPS is known to induce the expression of proinflammatory cytokines [27]. The mRNA levels of proinflammation-related genes were evaluated to determine the anti-inflammatory effects of the transgenic rice seed extracts. Treatment with the transgenic rice seed extracts significantly reduced the mRNA expression levels of proinflammation biomarkers in LPS-stimulated RAW264.7 cells (Figure 4). Moreover, the expression of the proinflammation-related genes was dramatically suppressed by pretreatment with transgenic rice seed extracts at 100 µg/mL. Compared with the treatment with DJ rice seed extracts, treatment with a low concentration of transgenic rice seed extracts (10 µg/mL) did not lead to a significant difference in cytokine expressions. However, compared with the treatment with DJ rice seed extracts, treatment with transgenic rice seed extracts at 100 µg/mL significantly decreased the expression of immune-related genes.

### 3.4. Effects on the mRNA Expression Levels of Immune-Associated Genes in RAW264.7 Cells

**Figure 5:**
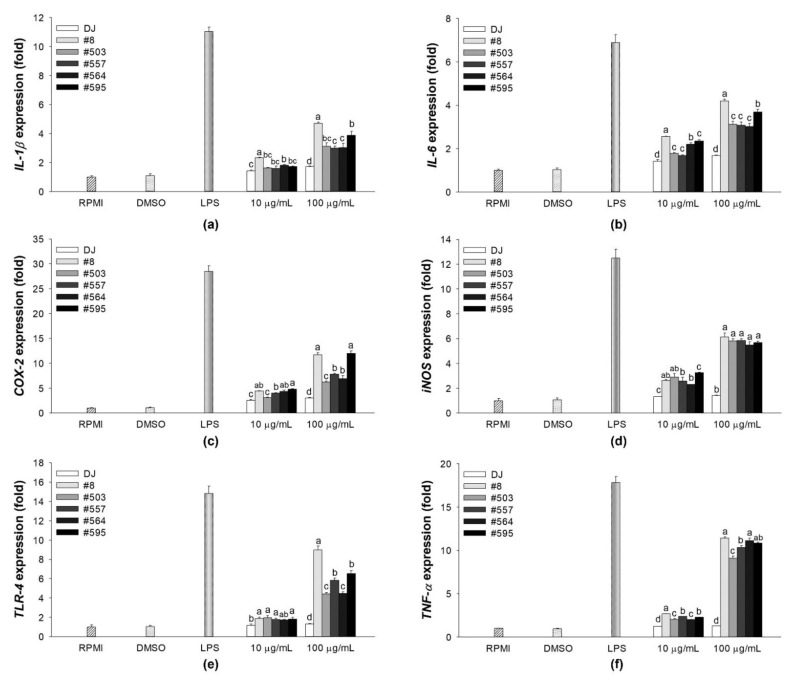
Effects of transgenic rice seed extracts on the expression of immune-associated genes in RAW264.7 cells without LPS stimulation. The expression levels of (**a**) *IL-1β*, (**b**) *IL-6*, (**c**) *COX-2*, (**d**) *iNOS,* (**e**) *TLR-4*, and (**f**) *TNF-α* expression levels are shown. The concentrations of DMSO and LPS were 0.1% and 1 µg/mL, respectively. Data are presented as means ± standard deviations. Lowercase letters (a, b, c, and d) indicate significant differences at *p* < 0.05 among treatments at the same concentration.

Treatment with transgenic rice seed extracts increased the expression levels of immune-associated genes in RAW264.7 cells. Treatment with 100 µg/mL of the transgenic rice seed extracts significantly increased the expression levels of proinflammatory cytokines (*IL-1β*, *IL-6*, and *TNF-α*) and inflammation biomarkers (*COX-2* and *iNOS*) (Figure 5). Interestingly, treatment with sample #8 at 100 µg/mL (not at 10 µg/mL) led to the highest increase in the expression of *TLR-4*, which encodes for a key receptor involved in LPS recognition [28]. These results indicated that compared with lower concentrations of transgenic rice seed extracts, higher concentrations led to a higher PPD production; a higher amount of PPD exerted higher immunomodulatory effects. Therefore, treatments with transgenic rice seed extracts at 100 µg/mL were used for further experiments.

### 3.5. Production of PGE_2_

**Figure 6:**
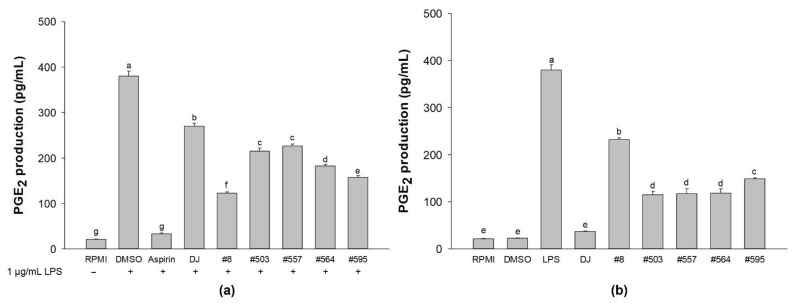
Effects of transgenic rice seed extracts (100 µg/mL) on PGE_2_ production. Effects on (**a**) RAW264.7 cells with LPS stimulation and (**b**) RAW264.7 cells without LPS stimulation. The concentrations of DMSO and aspirin were 0.1% and 200 µg/mL, respectively. Data are presented as means ± standard deviations. Lowercase letters (a, b, c, d, e, f, and g) indicate significant differences at *p* < 0.05 among the treatments.

PGE_2_ production increased when RAW264.7 cells were stimulated with LPS (Figure 6). However, pretreatments with the transgenic rice seed extracts led to the suppression of LPS-induced PGE_2_ production (Figure 6a). DJ rice seed extracts, treatment with transgenic rice seed extracts led to a markedly higher inhibitory effect on PGE_2_ production, particularly treatment with sample #8. No significant differences in PGE_2_ production were observed among the RPMI, DMSO, and DJ groups (Figure 6b). However, compared with the treatment with DJ rice seed extracts, treatment with transgenic rice seed extracts significantly enhanced the production of PGE_2_ in RAW264.7 cells.

### 3.6. Phagocytosis Activity

**Figure 7:**
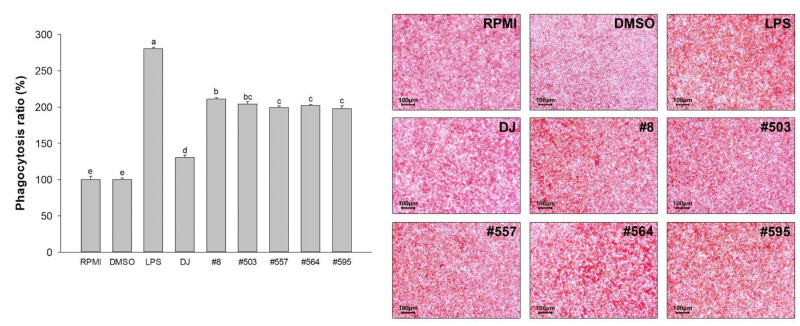
Effects of transgenic rice seed extracts (100 µg/mL) on phagocytosis activity in RAW264.7 cells. The concentrations of DMSO and LPS were 0.1% and 1 µg/mL, respectively. Data are presented as means ± standard deviations. Lowercase letters (a, b, c, d, and e) indicate significant differences at *p* < 0.05 among the treatments.

Treatment with LPS significantly increased the phagocytosis activity in RAW264.7 cells. Indeed, phagocytosis activity in the LPS-treated group was increased 2.80-fold compared with that in the nontreated (RPMI) group (Figure 7). Treatment with DJ and transgenic rice seed extracts resulted in the promotion of phagocytosis activity in RAW264.7 cells. Compared with the treatment with DJ rice seed extracts, pretreatment with the transgenic rice seed extracts increased phagocytosis activity in RAW264.7 cells, and the highest increase in activity was observed following treatment with sample #8 (1.62-fold increase).

### 3.7. Pathway Signaling

**Figure 8:**
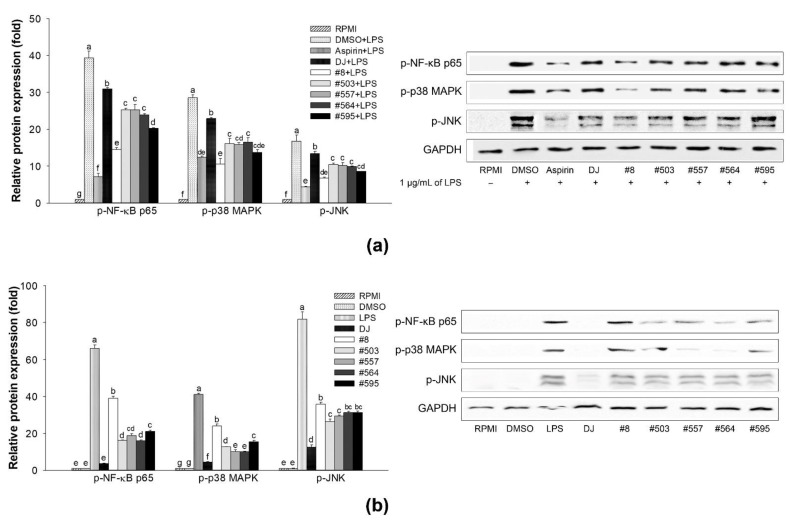
Effects of transgenic rice seed extracts (100 µg/mL) on the activation of the NF-κB and MAPK signaling pathways. Effects on (**a**) LPS-stimulated RAW264.7 cells and (**b**) RAW264.7 cells without LPS stimulation. The concentrations of DMSO and LPS were 0.1% and 1 µg/mL, respectively. Data are presented as means ± standard deviations. Lowercase letters (a, b, c, d, e, f, and g) indicate significant differences at *p* < 0.05 among the treatments.

To further assess the immunomodulatory effects of the transgenic rice seed extracts, the production of NF-κB- and MAPK-associated proteins was evaluated using a western blot assay. As shown in Figure 8, LPS activated the NF-κB and MAPK pathways via the upregulation of phosphorylated (p)-NF-κB p65, -p38 MAPK, and -JNK. Treatment with transgenic rice seed extracts significantly suppressed the production of LPS-induced phosphorylated proteins in LPS-stimulated RAW264.7 cells (Figure 8a). Moreover, compared with no treatment [nontreated (RPMI) group], treatment with transgenic rice seed extracts promoted the production of p-NF-κB p65, p-p38 MAPK, and p-JNK in RAW264.7 cells without LPS stimulation (Figure 8b). Similar to the effects of LPS, the upregulation of these proteins significantly activated the NF-κB and MAPK pathways. Treatment with sample #8 at 100 µg/mL led to the highest inhibition of p-NF-κB p65, p-p38 MAPK, and p-JNK production in LPS-stimulated RAW264.7 cells and caused the highest increase in the upregulation of these proteins in RAW264.7 cells without LPS treatment.

### 3.8. LC–MS Analysis

**Figure 9:**
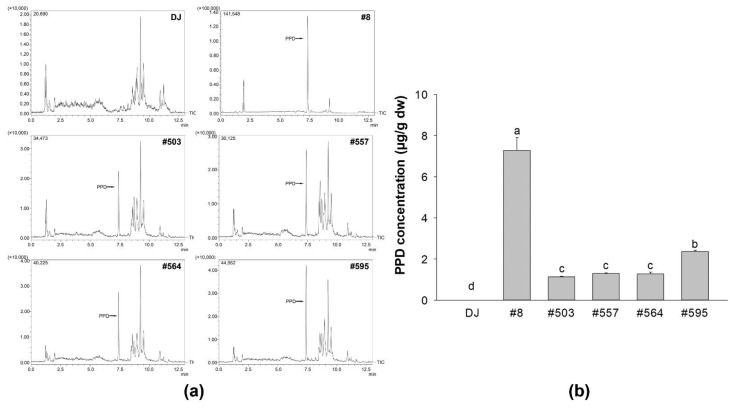
PPD production in the transgenic rice seed extracts. (**a**) Representation of the MS chromatograms of PPD detected in transgenic rice seed extracts (the Y axis of sample #8 is ×100,000, whereas the Y axes of the other samples are ×10,000). (**b**) The amount of PPD in each transgenic rice line. Data are presented as means ± standard deviations. Lowercase letters (a, b, c, and d) indicate significant differences at *p* < 0.05 among the treatments.

The amount of PPD in the transgenic rice seeds was quantified using LC–MS. The peak at the retention time of 7.344 min was the PPD signal used to identify PPD in the standard chromatogram. The peak at the retention time of 7.334 min was observed in the chromatograms of transgenic rice seed extracts but not in those of DJ extract (Figure 9a). The amount of PPD in the transgenic rice seeds from line #8 was significantly higher than that observed in the other transgenic lines (Figure 9b). The content of PPD in sample #8 was 7.28 ± 0.64 µg/g dry-weight, which was 3-fold higher than that in #595 and approximately 6-fold higher than that in #503, #557, and #564.

## 4. Discussion

Macrophages play important roles in protecting the body against harmful pathogens by facilitating immune responses [8]. The activation of macrophages is associated with the activation of immune-associated pathways. Activation of the MAPK and NF-κB signaling pathways leads to the production of immune-related cytokines and mediators (e.g., NO, *iNOS*, *COX-2*, PGE_2_, *IL-1β*, and *IL-6*) [29,30]. The release of NO directly damages foreign microorganisms [31,32]. IL-1β is secreted by activated macrophages to increase the viability of T cells and antigen-presenting activity [33]. TNF-α is a crucial element in the normal immune responses associated with the pathogenesis of various inflammatory diseases and enhancement of macrophage phagocytosis activity [34,35]. IL-6 promotes the differentiation of T cells and B cells [36] and increases phagocytosis activity [37]. In the present study, compared with no treatment, treatment with PPD-producing transgenic rice seed extracts significantly increased the production of immunomodulatory factors, such as NO (Figure 3), proinflammatory cytokines (Figure 5), and proinflammatory mediators (*COX-2* and PGE_2_; Figure 6b), in RAW264.7 cells. Moreover, treatment with 100 µg/mL of the transgenic rice seed extracts increased phagocytosis activity in RAW264.7 cells (Figure 7). Treatment with transgenic rice seed extracts enhanced the activation of macrophages through the MAPK and NF-κB pathways by increasing the phosphorylation of p38 MAPK, JNK, and NF-κB p65. Shin et al. [38] reported similar results, i.e., that heat-processed ginseng containing ginsenosides activated the MAPK and NF-κB signaling pathways, causing an increase in TNF-α and IL-6 production. Kim and Cho [21] reported that PPD upregulates the production of proinflammatory cytokines by activating the NF-κB and MAPK signaling pathways. In addition, PPD has been shown to affect the innate immune response by increasing the phagocytic uptake of macrophages [21,38].

LPS, which is a component of the outer membrane of gram-negative bacteria, is often used to mimic the inflammatory condition [39,40,41]. LPS triggering the innate immune response is recognized by TLRs. TLR-4 is reportedly an LPS receptor (see the review of Beutler [42]), and several studies have demonstrated that LPS activates the NF-κB and MAPK signaling pathways via TLR-4 [43,44,45]. In the present study, we showed that PPD-producing transgenic rice can suppress the activation of LPS-induced NF-κB and MAPK pathways in RAW264.7 cells. In the transgenic rice seed extract groups, the phosphorylation of NF-κB, p38 MAPK, and JNK was decreased significantly compared with that in the LPS treatment group (Figure 8a). This suppression led to the inhibition of NO (Figure 2) and PGE_2_ production (Figure 6a) in LPS-stimulated RAW264.7 cells, and the expression levels of immune-related mRNAs (*IL-1β*, *IL-6*, *TNF-α*, *iNOS*, and *COX-2*) were also downregulated in the transgenic rice seed extract treatment groups (Figure 4). These results are supported by the findings of Ahmmed et al. [46], who reported that PPD-type ginsenosides reduce NF-κB signaling pathway activity. In addition, Kim et al. [47] demonstrated the protective effects of PPD against LPS-induced inflammation, including the inhibition of NO production and proinflammatory enzyme expression (iNOS and COX-2). Furthermore, PPD treatment has also been shown to reduce the LPS-stimulated mRNA expression levels of *IL-1β*, *IL-6*, and *TNF-α* by blocking the activation of the NF-κB signaling pathway [48]. Similarly, PPD-enriched red ginseng extracts potently inhibit the mRNA expression of proinflammatory genes and proinflammatory mediator genes without causing cytotoxicity (up to 20 µg/mL) in LPS-stimulated RAW264.7 cells [49]. We found that treatment with sample #8 led to the highest change in immune-boosting effects in RAW264.7 cells and anti-inflammatory effects in LPS-stimulated RAW264.7 cells. This may be attributed to the high amount of PPD produced in transgenic rice line #8 (Figure 9), which was much higher than that produced in other transgenic rice lines. Consequently, the enhancement of immunity in RAW264.7 cells and inhibition of proinflammatory factors during LPS-induced inflammation are considered to be caused by the accumulation of PPD in transgenic rice seeds.

## 5. Conclusions

Our study demonstrated the anti-inflammatory and immune-boosting effects of PPD-producing transgenic rice seed extracts in RAW264.7 macrophage cells. Transgenic rice seed extracts induced macrophage activation through TLR-4-associated activation of the NF-κB and MAPK pathways in RAW264.7 cells. These activations led to a significant increase in NO production, phagocytosis activity, proinflammatory cytokine expression, and PGE_2_ production in RAW264.7 cells treated with transgenic rice seed extracts compared with their production in cells treated with normal rice seed extracts (DJ group). Moreover, the LPS-induced activation of the NF-κB and MAPK pathways was suppressed by treatment with the transgenic rice seed extracts. Depending on cell conditions, transgenic rice seeds containing PPD act as both stimulatory and anti-inflammatory agents. We suggest that PPD-producing transgenic rice could be developed and used as an immunomodulation agent following further research.

## Data Availability

All the applicable data have been provided in the manuscript. The authors will provide additional details if necessary.
